# The Association between VEGFR Gene Polymorphisms and Stroke: A Meta-Analysis

**DOI:** 10.1371/journal.pone.0151371

**Published:** 2016-03-16

**Authors:** Shi Qiu, Tao Wu, Peifu Wang, Jilai Li, Qin Li, Jichen Du

**Affiliations:** Department of Neurology, Aerospace Center Hospital, Beijing, China; National Cancer Center, JAPAN

## Abstract

Several published articles investigated the relationship between VEGF receptor gene polymorphisms and stroke, but they failed to reach the same conclusion. This meta-analysis was performed to identify the relationships between VEGF receptor gene and the risk of stroke. The PubMed, Embase, China National Knowledge Infrastructure (CNKI) database, Wanfang Chinese database, and VIP Chinese database were systemically searched. Data was extracted by two independent reviewers. The pooled odds ratio (OR) with 95% confidence interval (CI) were calculated. 5 case-control studies with a total of 2904 patients with stroke and 2824 control subjects were included, including 2904 cases and 2824 controls for -604T>C, 2733 cases and 2663 controls for +1192C>T, and 2733 cases and 2663 controls for +1719A>T. Under the dominant and recessive models, respectively, the overall ORs and 95% CIs of -604 C were 0.749, 0.493–1.138 (*P* = 0.176) and 0.819, 0.544–1.234 (*P* = 0.340); the overall ORs and 95% CIs of +1192 T were 1.148, 0.876–1.504 (*P* = 0.318) and 1.611, 1.004–2.586 (*P* = 0.048); the overall ORs and 95% CIs of +1719 T were 1.227, 0.932–1.615 (*P* = 0.146) and 1.139, 1.015–1.279 (*P* = 0.027). Our finding indicates that +1192C>T and +1719A>T may be associated with the risk of stroke, but not -604T>C.

## 1. Introduction

Stroke is one of the most complex diseases with diverse etiologies. It is well established that genetic and environmental backgrounds play a crucial role in the pathogenesis of stroke[1]. Environmental factors, such as smoking, hypertension and diabetes mellitus may contribute to the development of stroke. Currently, several candidate genes have been linked to stroke in genome-wide association studies, but the contribution of susceptibility genes to stroke is still obscure[2].

Vascular endothelial growth factor (VEGF) plays an important role in the maintenance of endothelial integrity, endothelial survival and the physiological function of endothelium. Emerging evidence suggests that polymorphism of the VEGF gene may be associated with the risk of stroke and other cerebral vascular disease[3–6]. The bioactivity of VEGF is mediated by two receptor tyrosine kinases, VEGF receptor-1 (VEGFR1, also called Flt-1) and VEGF receptor-2 (VEGFR2, also called kinase insert domain-containing receptor, KDR). Of the two VEGFR isoforms, KDR is the main receptor and plays a pivotal role in endothelial integrity and function[7, 8]. Several single nucleotide polymorphisms (SNPs) of KDR were found in the promoter region and coding region. Three of them were believed to affect the activity of VEGF-KDR signaling pathway and investigated the most frequently: +1192C>T (rs2305948), +1719A>T (rs1870377) and -604T>C (rs2071559).+1192C>T and +1719A>T are both found in exon regions of VEGF receptor-2 and lead to amino acid substitutions that reduce the binding affinity of VEGF to VEGF receptor-2. -604T>C is located in the promoter region and leads to decreased promoter activity.

Many genetic association studies have been carried out to assess the relationship of these three variants with stroke. However, the findings of them remain controversial. Up to now, no meta-analysis has been performed to investigate this relationship. Therefore, we conduct this meta-analysis to deal with these contradictory results and assess whether VEGF receptor-2 polymorphisms contribute to the risk of stroke.

## 2. Methods

### 2.1 Search strategy

Publication search was performed for the potential eligible articles in English and Chinese in the following database: (1) Medline in PubMed searching engine; (2) Embase database; (3) Chinese National Knowledge Infrastructure (CNKI); (4) Wanfang Chinese database; (5) VIP Chinese database. The latest data for searching articles was November 1^st^, 2015. The key words for article searching were: [“vascular endothelial growth factors receptor” or “vasculotropin receptor” or “VEGFR”] and [“stroke” or “cerebral infarction” or “cerebrovascular disorders”] and [“single nucleotide polymorphism” or “SNP” or “polymorphism” or “mutation” or “genetics” or “variant”]. Publication language was restricted to English and Chinese, and the subjects were not limited in our search. We also performed a manual search of the reference lists of retrieved articles for additional potential studies.

### 2.2 Inclusion criteria

The inclusion criteria for the gene association studies in this meta-analysis were as follows: (1) independently published case-control studies explored the association between VEGF receptor gene polymorphisms and stroke; (2) with genotype or allelic distributions provided; (3) with data in any of the three polymorphisms, and sufficient data available to calculate an odd ratio (OR) with its 95% confidential interval (CI); (4) if the authors published two or more studies using the same subjects, the most recent publication or the publication with the largest sample size was include. No limitations were placed on race, ethnicity, or geography area.

### 2.3 Data extraction

Relevant data were systematically extracted from the included studies by two authors using a standardized form, and reached a consensus on all items. The researchers collected the following data: the first author’s name, publication year, countries and ethnicities of participants, sample size, and genotyping method.

### 2.4 Quality score assessment

To determine the methodological quality of the included studies, we used the Newcastle-Ottawa scale (NOS)[9] to judge the quality of these case-control studies. The NOS ranges from zero to nine stars, and a score ≥ 7 was considered to be of high quality. Two authors assessed the quality of included studies independently, and all disagreements were resolved by discussion.

### 2.5 Evaluation of statistic association

The association between -604T>C, +1192C>T, and +1719A>T polymorphisms and the risk of stroke was tested by calculating OR and 95% CI. The dominant and recessive models were applied for the genotype comparison. Heterogeneity between studies was estimated by Cochran’s χ^2^ based Q-statistic test[10] and *I*^2^ test. The heterogeneity was considered to be statistically significant at *P*≤0.1 or *I*^2^ >50%. When the *P* value was >0.1 and *I*^2^ ≤50%, the pooled OR was calculated by fixed-effects model, otherwise, the random-effects was applied. Pooled ORs were calculated by the method of Mantel-Haenszel and DerSimonian-Laird, respectively. 95% CI was estimated by Woolf’s method. Publication bias was explored using funnel plots and Egger’s regression test (*P*<0.05 indicated statistical significance)[11]. Hardy-Weinberg equilibrium (HWE) of the genotype distribution of controls was conducted by Pearson’s χ^2^ test. Sensitivity analysis was performed by limiting the meta-analysis to case-control studies with high quality (NOS score ≥ 7). All statistic tests were conducted by Stata 11.0 software.

## 3. Results

### 3.1 Included studies

**Fig 1:**
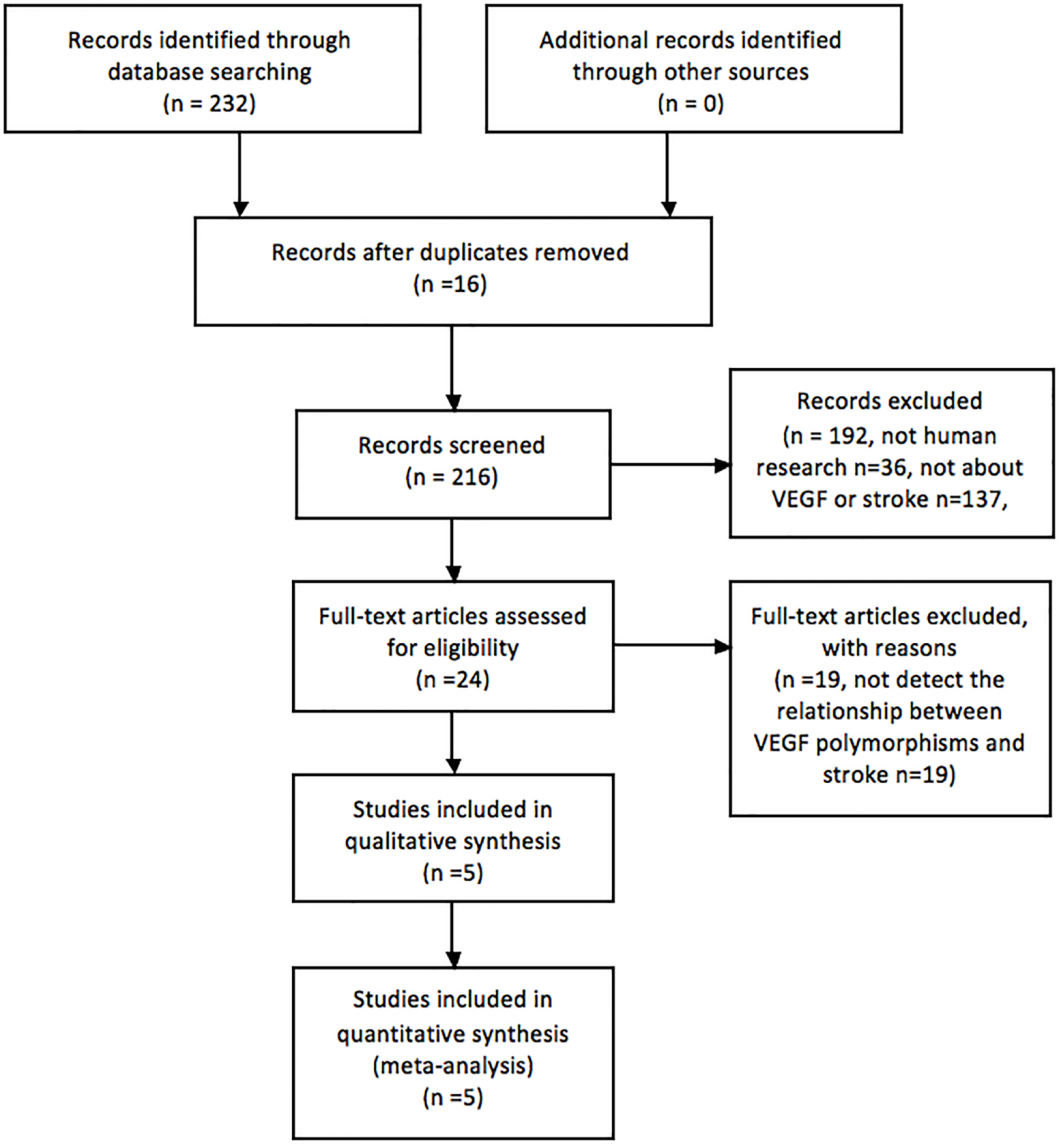
Flow chart of the literature search and selection.

Fig 1 showed the process of retrieving eligible studies. Initially, our highly sensitive search strategy identified 216 articles. After reviewed the titles and abstracts of all articles, 192 articles were excluded. After systematically reading full texts, we excluded another 19 articles. Finally, 5 case-control studies with a total of 2904 patients with stroke and 2824 control subjects met our inclusion criteria for qualitative data analysis[12–16]. Table 1 summarized the characteristics of the studies included in the meta-analysis. 5 studies with 2904 cases and 2824 controls for -604T>C, 3 studies with 2733 cases and 2663 controls for +1192C>T and 3 studies with 2733 cases and 2663 controls for +1719A>T were selected eventually. Table 2 showed the studies that have provided the distribution of VEGF receptor-2 genotype and allele among stroke patients and controls. The NOS results showed that the average score was 7.8, which indicated that the methodological quality of included studies was generally good.

**Table 1:** Characteristics of studies included in the meta-analysis.

Author	Year	Country	Ethnicity	Male (%)	Age (year)	Sample size		Genotype method	Polymorphism	NOS score
						Case	Control			
Zhang et al.	2009	China	Asian	63.4	60.4±9.2	1849	1798	PCR-RFLP	-604T/C, +1192C/T, +1719A/T	9
Oh et al.	2011	Korea	Asian	58.7	63.0±11.4	501	478	PCR-RFLP	-604T/C, +1192C/T, +1719A/T	8
Han et al.	2012	Korea	Asian	43.1	62.4±12.0	383	387	PCR-RFLP	-604T/C, +1192C/T, +1719A/T	8
Shen et al.	2014	China	Asian	67.0	63.2±10.9	103	43	PCR-RFLP	-604T/C	8
Zhang et al.	2014	China	Asian	51.2	57.6±10.1	68	118	PCR-RFLP	-604T/C	6

**Table 2:** Distribution of VEGF receptor-2 genotype and allele among stroke patients and controls in three SNPs.

Author	Sample size		-604T/C						+1192C/T						+1719A/T					
			T	C	TT	TC	CC	HWE	C	T	CC	CT	TT	HWE	A	T	AA	AT	TT	HWE
Zhang et al. (2009)	Case	1849	2590	1108	920	750	179		3183	515	1367	449	33		2248	1450	709	830	310	
	Control	1798	2475	1121	862	751	185	0.26	3209	387	1429	351	18	0.49	2215	1381	699	817	282	0.09
Oh et al. (2011)	Case	501	696	306	236	224	41		874	128	381	112	8		500	502	119	262	120	
	Control	478	678	278	241	196	41	0.90	851	105	378	95	5	0.72	554	402	159	236	83	0.78
Han et al. (2012)	Case	383	555	211	202	151	30		685	81	307	71	5		414	352	113	188	82	
	Control	387	591	183	229	133	25	0.34	681	93	299	83	5	0.78	443	331	129	185	73	0.64
Shen et al. (2014)	Case	103	185	21	86	13	4													
	Control	43	47	39	14	19	10	0.47												
Zhang et al. (2014)	Case	68	102	34	39	24	5													
	Control	134	163	73	57	49	12	0.76												

### 3.2 Association of VEGFR2 polymorphisms and stroke

**Fig 2:**
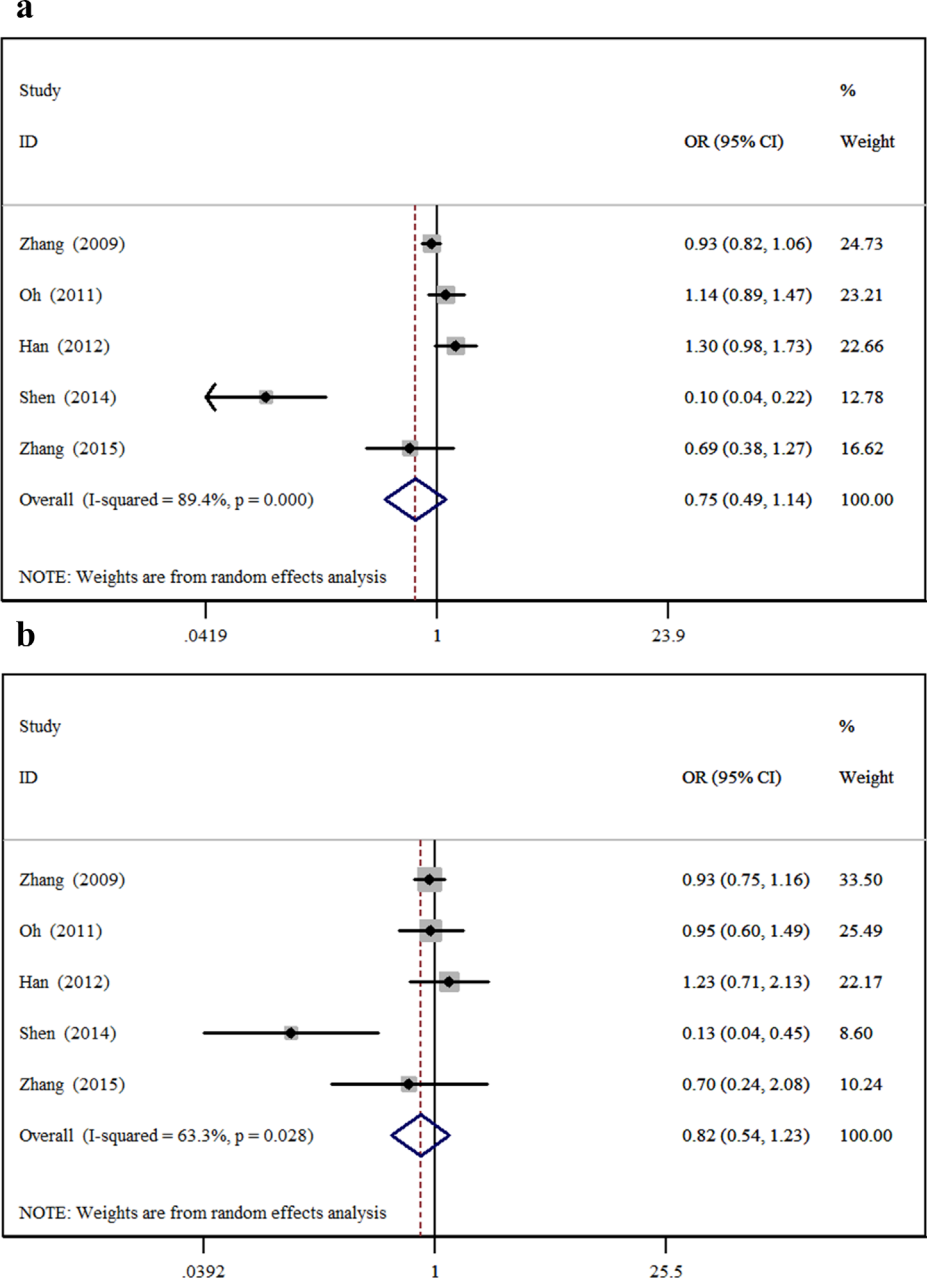
The association between -604T>C and stroke in different genetic models. **a** Dominant model. **b** Recessive model.

**Fig 3:**
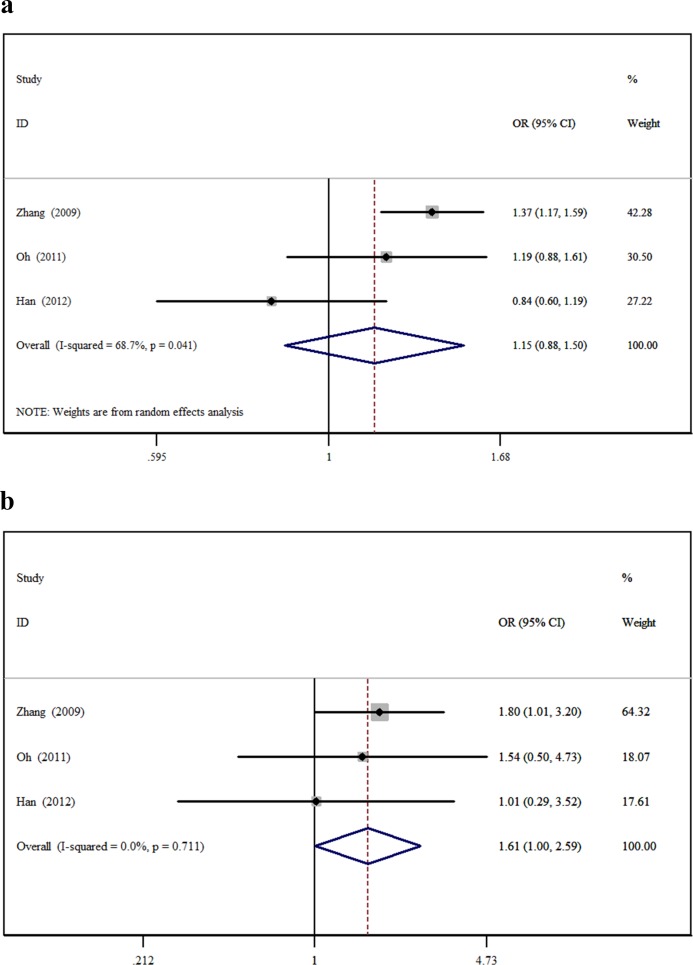
The association between +1192C>T and stroke in different genetic models. **a** Dominant model. **b** Recessive model.

**Fig 4:**
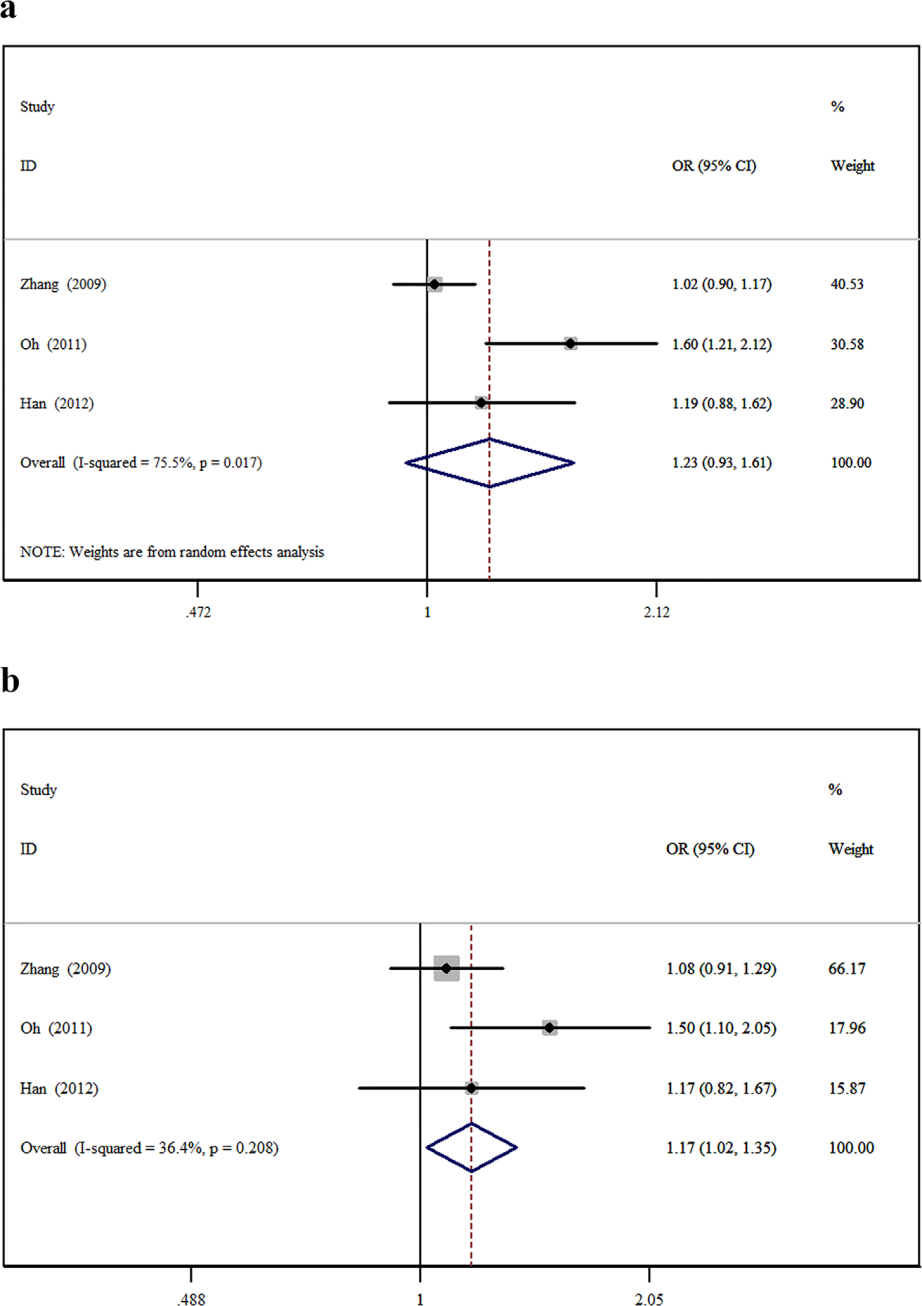
The association between +1719A>T and stroke in different genetic models. **a** Dominant model. **b** Recessive model.

The minor allele and major allele were compared in the dominant and recessive models. The overall ORs and 95% CIs of -604 C were 0.749, 0.493–1.138 (*P* = 0.176) and 0.819, 0.544–1.234 (*P* = 0.340) compared with T in the dominant and recessive models, respectively (Fig 2). The overall ORs and 95% CIs of +1192 T were 1.148, 0.876–1.504 (*P* = 0.318) and 1.611, 1.004–2.586 (*P* = 0.048) compared with C in the dominant and recessive models, respectively (Fig 3). The overall ORs and 95% CIs of +1719 T were 1.227, 0.932–1.615 (*P* = 0.146) and 1.139, 1.015–1.279 (*P* = 0.027) compared with A in the dominant and recessive models, respectively (Fig 4) (Table 3).

**Table 3:** The association between VEGF receptor-2 gene polymorphisms and stroke in different genetic models

Gene polymorphism	Number of studies	Genetic model	OR	95% CI	*P* value
-604T>C	5	Dominant	0.749	0.493–1.138	0.176
		Recessive	0.819	0.544–1.234	0.340
+1192C>T	3	Dominant	1.148	0.876–1.504	0.318
		Recessive	1.611	1.004–2.586	0.048
+1719A>T	3	Dominant	1.227	0.932–1.615	0.146
		Recessive	1.172	1.018–1.349	0.027

### 3.3 Sensitivity analysis

**Fig 5:**
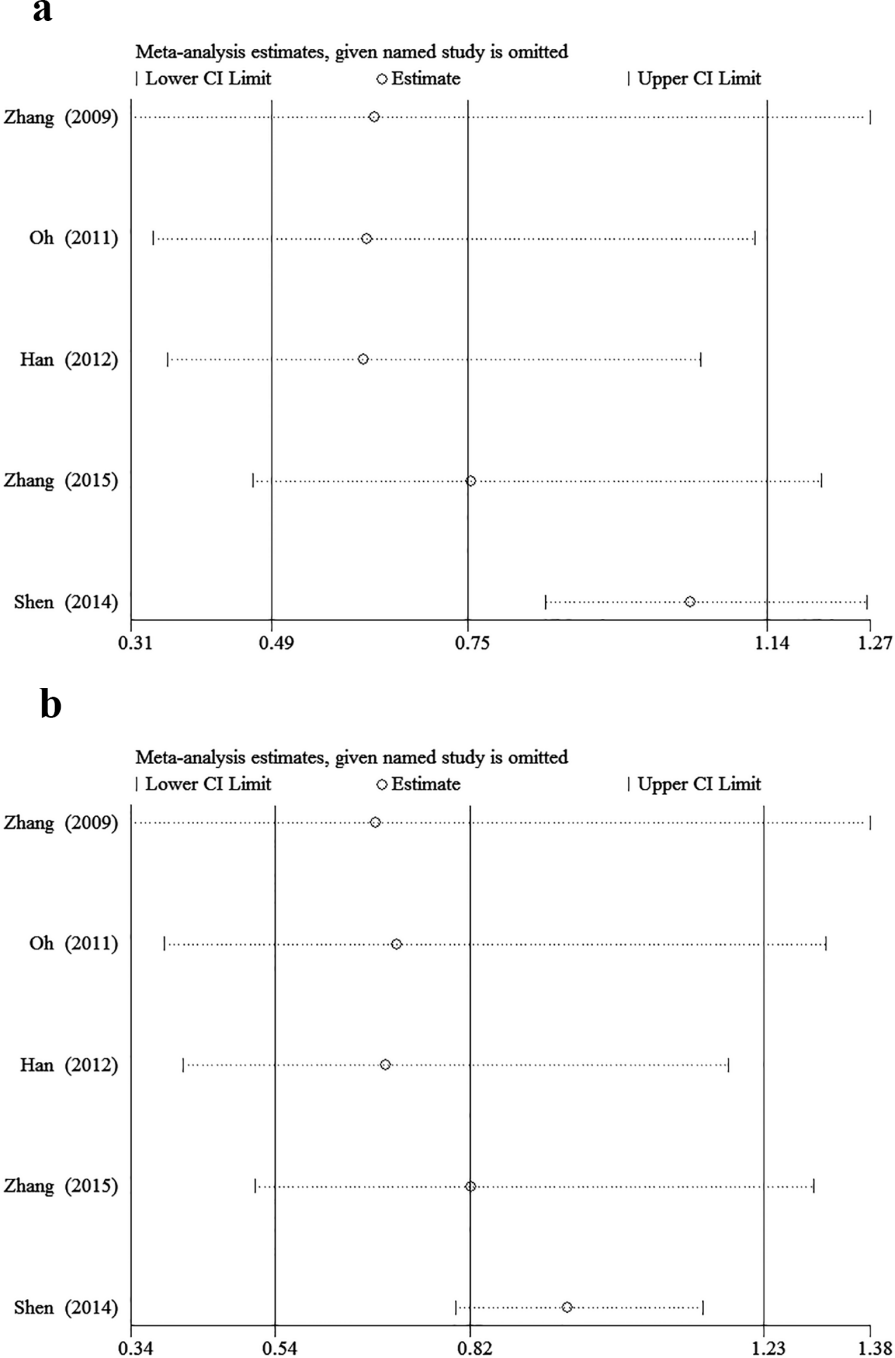
Sensitivity analysis of the summary OR coefficients on the association between -604T>C and stroke in different genetic models. **a** Dominant model. **b** Recessive model.

**Fig 6:**
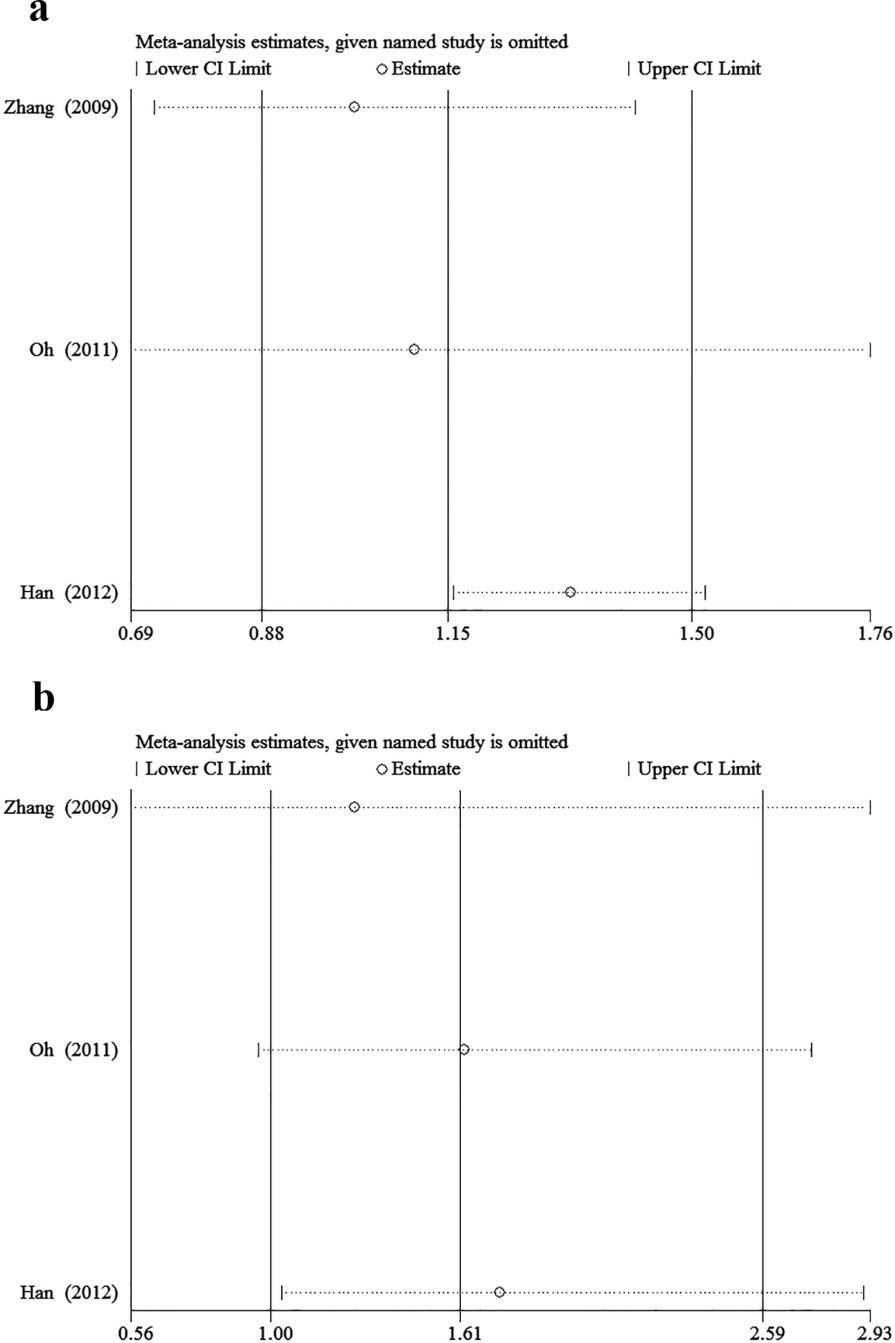
Sensitivity analysis of the summary OR coefficients on the association between +1192C>T and stroke in different genetic models. **a** Dominant model. **b** Recessive model.

**Fig 7:**
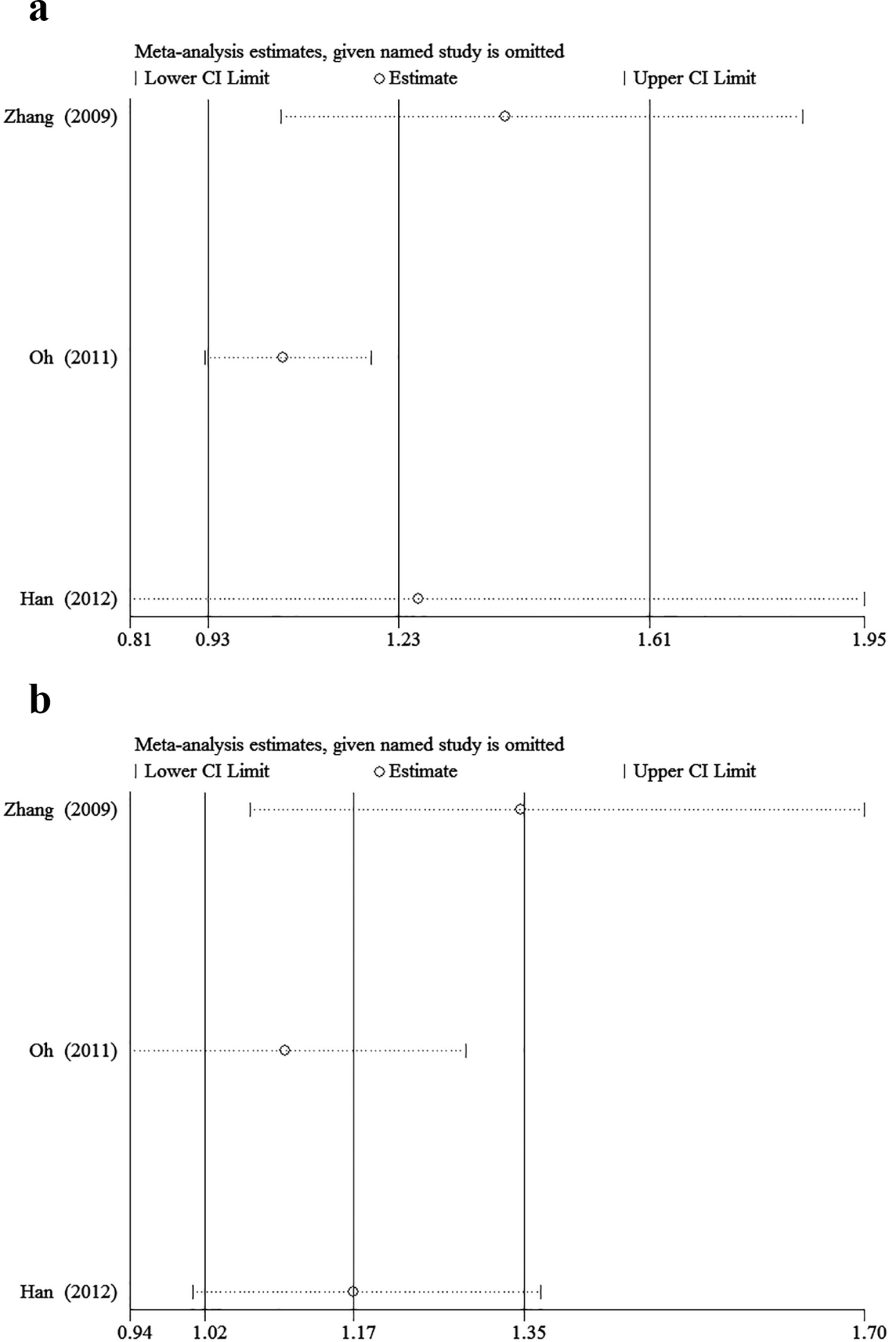
Sensitivity analysis of the summary OR coefficients on the association between +1719A>T and stroke in different genetic models. **a** Dominant model. **b** Recessive model.

Sensitivity analysis was performed to assess the stability of results. Sensitivity analysis of the summary odds ratio coefficients on the relationships of the three SNPs and the risk of stroke is computed by omitting each study in turn(Figs 5–7). Furthermore, by limiting the meta-analysis to case-control studies with high quality (NOS score ≥ 7), the sensitivity analysis was conducted in another way. As a result, we omitted one study[16] in the comparison of -604T>C. However, the corresponding ORs were not substantially altered in comparisons, indicating that our results were relatively robust. The results of sensitivity analysis were shown in Table 4.

**Table 4:** Sensitivity analysis of VEGF receptor-2 gene -604T>C polymorphism and stroke risk.

Category	Numbers	Sample size		Dominant model				Recessive model			
		Case	Control	OR	95% CI	*P*	*I*^2^	OR	95% CI	*P*	*I*^2^
Overall	5	2904	2824	0.75	0.49–1.14	0.18	89.40%	0.82	0.54–1.23	0.34	63.30%
SA	4	2836	2706	0.75	0.47–1.21	0.24	91.80%	0.82	0.52–1.30	0.40	71.90%

SA: sensitivity analysis based on NOS score (studies with NOS score ≥ 7 were included)

### 3.4 Publication bias

**Fig 8:**
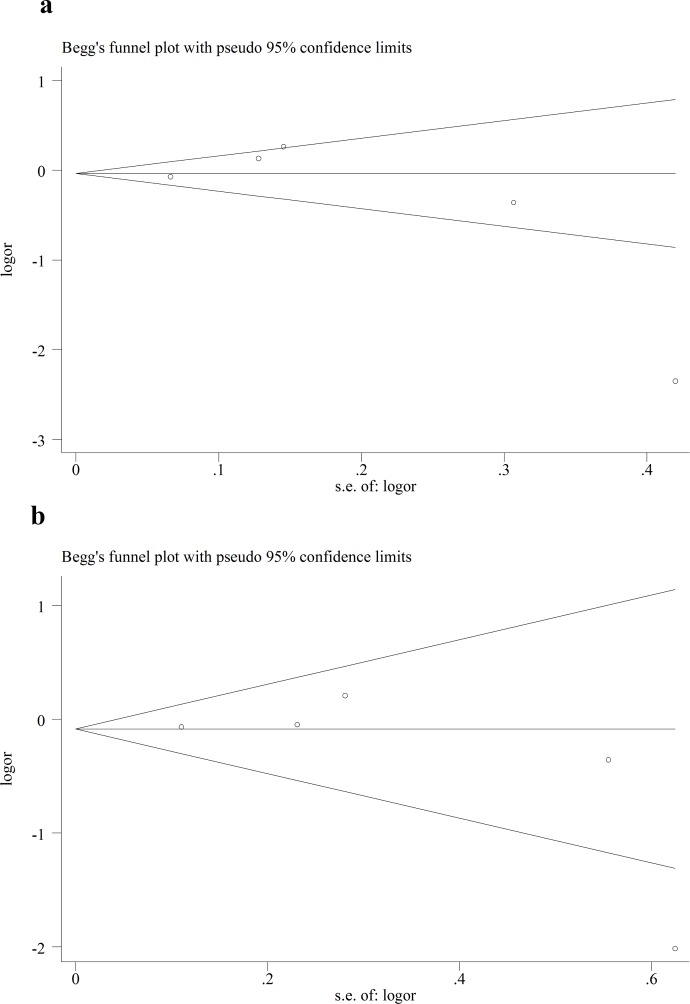
Egger’s funnel plot in assessing publication bias about -604T>C and stroke in different genetic models. **a** Dominant model. **b** Recessive model.

**Fig 9:**
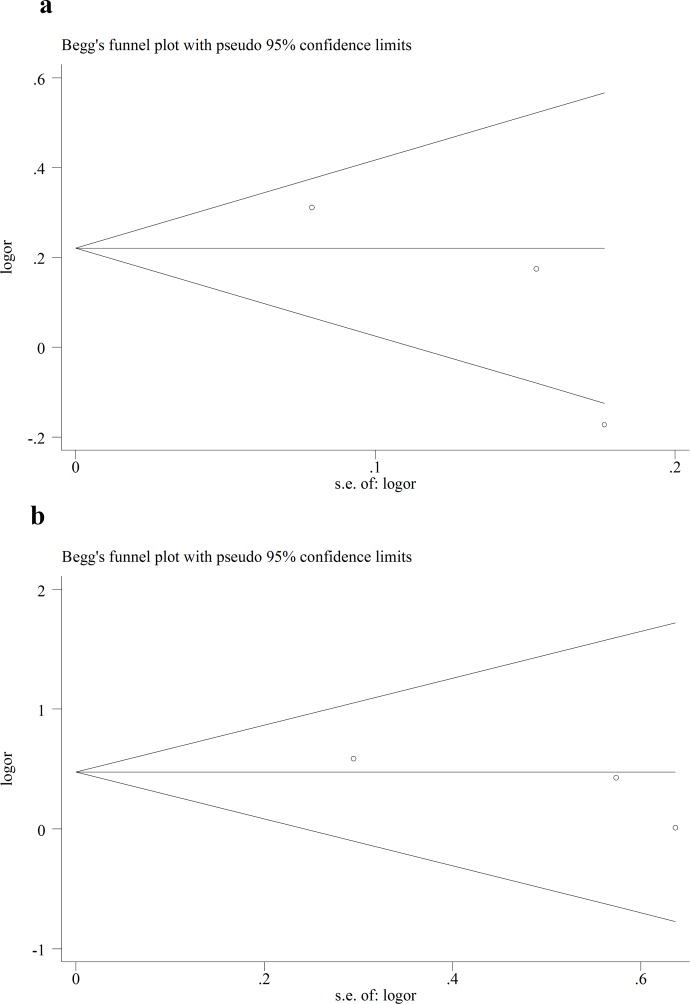
Egger’s funnel plot in assessing publication bias about +1192C>T and stroke in different genetic models. **a** Dominant model. **b** Recessive model.

**Fig 10:**
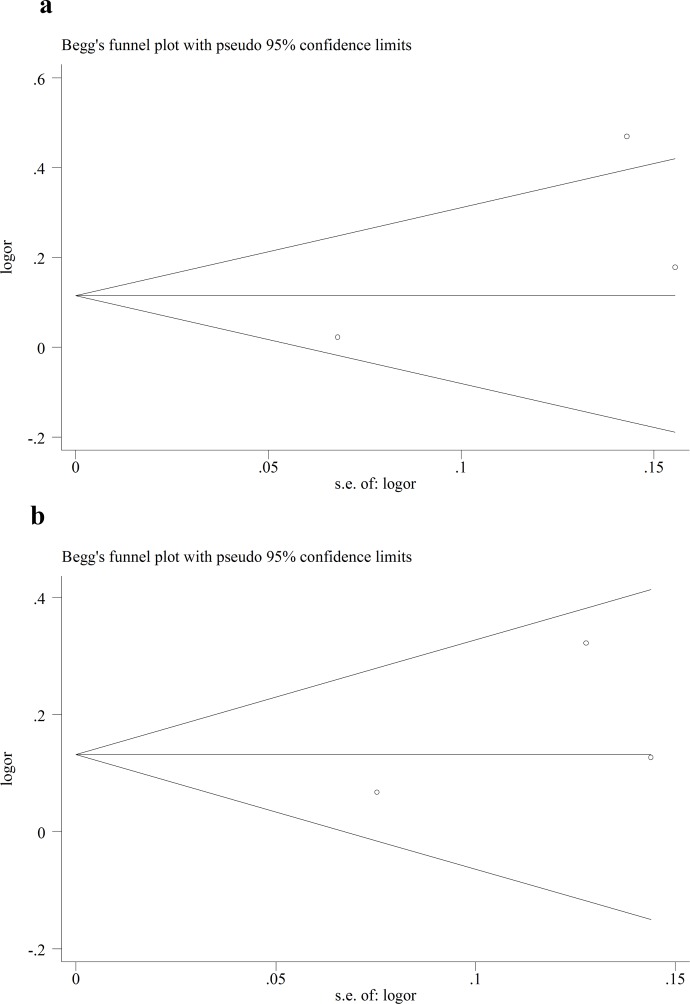
Egger’s funnel plot in assessing publication bias about +1719A>T and stroke in different genetic models. **a** Dominant model. **b** Recessive model.

We performed Funnel plot and Egger’s linear regression test to detect publication bias. The shape of funnel plot looks symmetrical for all the comparison models, and the Egger’s test was used to provide statistical evidence of publication funnel plot symmetry. No obvious evidence of publication bias was revealed by the results (Figs 8–10) (Table 5).

**Table 5:** Egger’s linear regression test to measure the funnel plot asymmetric.

Polymorphism	Comparisons	Study	*t*	*P* value	95% CI
-604T>C	Dominant	Overall	-1.01	0.39	-10.7–5.55
	Recessive	Overall	-1.12	0.34	-5.57–2.67
+1192C>T	Dominant	Overall	-1.98	0.30	-27.38–19.99
	Recessive	Overall	-1.79	0.32	-9.77–7.36
+1719A>T	Dominant	Overall	1.47	0.38	-27.91–35.23
	Recessive	Overall	0.97	0.51	-29.23–34.06

## 4. Discussion

We conducted the meta-analysis to investigate the relationship between the three SNPs of VEGF receptor-2 gene and stroke risk. To our knowledge, this is the first time. The main finding was that +1192 T and +1719 T may confer risk of stroke in the recessive model. However, our data revealed no association between -604T>C polymorphisms and stroke risk.

VEGF receptor-2, also called KDR, is the main receptor for VEGF. VEGF-KDR signaling pathway plays a critical role in the development of vascular disease by impacting survival, proliferation, and migration of endothelial cell[17]. Animal researches have shown that deficiency of VEGF receptor-2 gene could result in abnormal blood vessels and defective development of endothelial cell[18]. Evidence suggests that all the three SNPs of VEGF receptor-2, -604T>C, +1192C>T, and +1719A>T, have a significant association with the development of coronary heart disease[19].

It remains debatable how the VEGF signaling pathway affect the pathogenesis of stroke. Evidences support the involvement of angiogenesis in stroke. Vascular endothelial growth factors (VEGF) have important roles in the development and function of the circulation system, which have been shown to participate in atherosclerosis and angiogenesis[5]. On the one hand, several studies suggest that increased VEGF signaling aggravates atherosclerosis through neovascularization and inflammation in atheromatous plaques. Increased density of microvessels within the plaque contributes to the growth and destabilization of the plaque, resulting in the narrowing and occlusion of large cerebral arteries[20, 21]. On the other hand, the lack of sufficient VEGF signaling could result in endothelial dysfunction, vascular degeneration, and formation of weak, thin walled vasculature, which can reduce vessel compliance and increase the risk of spontaneous vessel wall rupture[22, 23]. VEGF receptor-2 is the main receptor for VEGF. To investigate whether the polymorphisms of VEGF receptor-2 are associated with the risk of stroke, may contribute to the study of the mechanisms of stroke.

Although there are plenty of studies investigating the relationship between the three SNPs of VEGFR2 and stroke risk, their results were inconsistent or even contradictory. That is the reason we conduct this meta-analysis. In our results, we found two of the three SNPs might be associated with stroke risk, which was not gained by any of the original articles. We postulate that the sample size of the original articles is relatively small and not sufficient enough to get a conclusive result. In the present meta-analysis, +1192C>T and +1719A>T SNPs were associated with the risk of stroke, but not -604T>C. These results may be attributed to the different locations of the three SNPs in the VEGF receptor-2 gene. SNP +1192C>T is located in exon 7 and +1719A>T is located in exon 11, which lead to amino acid substitutions, Val297Ile and Gln472His, respectively, and reduce binding affinity of VEGF to VEGF receptor-2. However, -604T>C is located in the promoter region of VEGF receptor-2 gene, which may lead to a decreased expression of VEGF receptor-2. It is obscure whether the amount or the binding affinity of VEGF receptor-2 plays a more important role in the VEGF-KDR pathway. Nevertheless our finding suggested that the binding affinity of VEGF receptor-2 affected by +1192C>T and +1719A>T might play a more critical role in the function of VEGF.

In addition, genotype distributions of controls in all studies were consistent with HWE. Sensitivity analysis was also performed, and it didn’t have significant impact on the combined ORs. In the present studies, Funnel plot and Egger’s linear regression test were conducted, but no publication bias was found. This made the results of this meta-analysis more reliable to some extent.

However, there remained some limitations in this meta-analysis. First, the number of studies enrolled in this meta-analysis was relatively small, which makes it hard to perform subgroup analysis and the analysis of different stroke subtypes. Second, all included articles were published in English or Chinese. Therefore, studies issued in other languages might be missed. Third, although the genotyping methods in all studies were the same, other factors like age, sex might lead to bias, which would need further investigation.

In conclusion, our study suggests that +1192 T and +1719 T in the VEGF receptor-2 gene may be associated with an increased risk of stroke. No association with stroke risk is identified in -604T>C polymorphisms. Due to the limitations mentioned above, further researches are required to confirm the findings.

## Supporting Information

S1 Diagram PRISMA 2009 flow diagram. (DOC)

S1 Checklist PRISMA 2009 checklist. (DOC)

S2 Checklist meta-analysis-on-genetic-association-studies-form. (DOCX)
