# Supplementary material for: The Association between VEGFR Gene Polymorphisms and Stroke: A Meta-Analysis
Source: PLoS One. 2016 Mar 16;11(3):e0151371. doi: 10.1371/journal.pone.0151371 (PMC4794216; doi:10.1371/journal.pone.0151371)
Supplement: S1 Diagram — (DOC) [file pone.0151371.s001.doc]

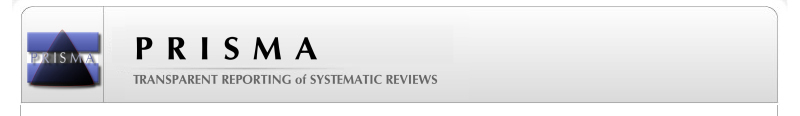
**PRISMA 2009 Flow Diagram**

**Screening**

**Included**

**Eligibility**

**Identification**

Records identified through database searching
(n = 232)

Additional records identified through other sources
(n = 0)

Records after duplicates removed
(n =16)

Records screened
(n = 216)

Records excluded
(n = 192, not human research n=36, not about VEGF or stroke n=137, review article n=19)

Full-text articles assessed for eligibility
(n =24)

Full-text articles excluded, with reasons
(n =19, not detect the relationship between VEGF polymorphisms and stroke n=19)

Studies included in qualitative synthesis
(n =5)

Studies included in quantitative synthesis (meta-analysis)
(n =5)
